# A Multifaceted Role of Tryptophan Metabolism and Indoleamine 2,3-Dioxygenase Activity in *Aspergillus fumigatus*–Host Interactions

**DOI:** 10.3389/fimmu.2017.01996

**Published:** 2018-01-22

**Authors:** Tsokyi Choera, Teresa Zelante, Luigina Romani, Nancy P. Keller

**Affiliations:** ^1^Department of Medical Microbiology and Immunology, University of Wisconsin-Madison, Madison, WI, United States; ^2^Department of Experimental Medicine, University of Perugia, Perugia, Italy; ^3^Department of Bacteriology, University of Wisconsin-Madison, Madison, WI, United States

**Keywords:** *Aspergillus fumigatus*, tryptophan metabolism, IDO, kynurenines, toxins, non-ribosomal peptides, peripheral tolerance, Th17 cells

## Abstract

*Aspergillus fumigatus* is the most prevalent filamentous fungal pathogen of humans, causing either severe allergic bronchopulmonary aspergillosis or often fatal invasive pulmonary aspergillosis (IPA) in individuals with hyper- or hypo-immune deficiencies, respectively. Disease is primarily initiated upon the inhalation of the ubiquitous airborne conidia—the initial inoculum produced by *A. fumigatus*—which are complete developmental units with an ability to exploit diverse environments, ranging from agricultural composts to animal lungs. Upon infection, conidia initially rely on their own metabolic processes for survival in the host’s lungs, a nutritionally limiting environment. One such nutritional limitation is the availability of aromatic amino acids (AAAs) as animals lack the enzymes to synthesize tryptophan (Trp) and phenylalanine and only produce tyrosine from dietary phenylalanine. However, *A. fumigatus* produces all three AAAs through the shikimate–chorismate pathway, where they play a critical role in fungal growth and development and in yielding many downstream metabolites. The downstream metabolites of Trp in *A. fumigatus* include the immunomodulatory kynurenine derived from indoleamine 2,3-dioxygenase (IDO) and toxins such as fumiquinazolines, gliotoxin, and fumitremorgins. Host IDO activity and/or host/microbe-derived kynurenines are increasingly correlated with many *Aspergillus* diseases including IPA and infections of chronic granulomatous disease patients. In this review, we will describe the potential metabolic cross talk between the host and the pathogen, specifically focusing on Trp metabolism, the implications for therapeutics, and the recent studies on the coevolution of host and microbe IDO activation in regulating inflammation, while controlling infection.

## Introduction

*Aspergillus fumigatus* is a saprophytic fungus that has a worldwide distribution. The asexual spores (called conidia) are ubiquitous and individuals inhale hundreds of spores daily. While most inhaled conidia are cleared by individuals with a healthy immune system, *A. fumigatus* can act as an opportunistic human pathogen in individuals with altered immune functions. Disease presentation can vary on the status of the host’s immune system; *A. fumigatus* can cause allergic bronchopulmonary aspergillosis, a severe allergenic response, in the hyper-immune, or the fatal invasive growth invasive pulmonary aspergillosis (IPA) in the hypo-immune, or in individuals with other susceptibilities such as patients unable to mount the necessary oxidative defenses such as in individuals with chronic granulomatous disease (CGD) (1).

The manifestation of disease is dependent not only on the host’s immune status but also fungal factors including strain heterogeneity (2). *A. fumigatus* growth in the mammalian lung, following survival of resident pulmonary defenses, requires the fungus to adapt to a hypoxic and nutritionally scarce environment. *Aspergillus* mutants unable to synthesize primary metabolites necessary for growth are generally impaired in virulence. For example, deletion of *cpcA*, a transcription factor that globally modulates amino acid biosynthesis in the fungus led to a less virulent phenotype in a murine model of IPA (3). Additional studies have shown that mutants in sulfur utilization (4), uracil/uridine synthesis (5), zinc uptake, iron acquisition, and many more (6, 7) are also decreased in virulence.

To complicate disease progression further, there is an alarming rise in antifungal resistance strains of *A. fumigatus* (8, 9). Therefore, an understanding of *A. fumigatus* and host metabolic pathways is important in identifying nutrient limitations. One critical metabolic pathway is the biosynthesis of aromatic amino acids [AAAs, tryptophan (Trp), phenylalanine, and tyrosine], which are required not only for growth of *A. fumigatus* but are also precursors for several toxins (Table 1). The host relies on dietary sources for all AAAs while *A. fumigatus* synthesizes all three. However, the host and *A. fumigatus* both possess AAA catabolic enzymes. In particular, one key enzyme important in immune homeostasis is indoleamine 2,3-dioxygenase (IDO), which converts Trp to kynurenine and related metabolites in both organisms. Historically, host IDOs activity has been described as an effective antimicrobial control for pathogens that are natural Trp auxotrophs such as *Staphylococcus aureus, Chlamydia* spp., and *Toxoplasma gondii*, presumably by Trp starvation (10). However, *A. fumigatus* can synthesize its own Trp and thus the Trp starvation may not be an effective pathogen control for those microbes able to synthesize their own Trp pools. Although, IDOs also play additional roles in host defenses through modifying kynurenine levels and subsequent cytokine responses as described below. In this review, we will summarize the recent studies describing the anabolic and the catabolic pathways of Trp metabolism, the implications for therapeutics, and the host–pathogen interaction.

**Table 1:** *Aspergillus fumigatus* non-ribosomal peptides containing aromatic amino acids (AAAs) in their peptide structure.

Toxin	AAA^a^	Interaction with host^b-i^
Fumigaclavine C^b^	Tryptophan (Trp)	Downregulation of Th1 cytokines including TNF-α, IL-1β, and IL-17A. Induction of host cell apoptosis Decrease activation of caspase-1
Fumiquinazoline C^c^	Anthranilate Trp	Cytotoxic
Fumisoquin^d^	Tyrosine	Nothing reported
Fumitremorgin^e^ Tryprostatin A^f^ Verruculogen^g^	Trp	Neurotoxic and produces tremors in mice Tryprostatin causes the inhibition of microtubule assembly Verruculogen causes modification of the electrophysiological properties of HNEC
Gliotoxin^h^	Phenylalanine	Virulent in a steroid murine model of IPA Induction of host cell apoptosis and causes epithelial cell damage Inhibition of phagocytosis and oxidative bursts
Hexadehydroastechrome^i^	Trp	Overexpression resulted in a significantly higher virulence in a neutropenic murine model of IPA^b^

*IPA, invasive pulmonary aspergillosis; HNEC, human nasal epithelial cells*.

*^a^Only the AAA is designated. Other amino acids are also in the structure of these metabolites*.

*^b–i^Biosynthesis and host interactions based from the following sources: ^b^(11, 12); ^c^(13); ^d^(14); ^e^(15, 16); ^f^(17); ^g^(18, 19); ^h^(7, 20); ^i^(21, 22)*.

## Trp Synthesis and Potential Therapeutic Targeting

**Figure 1:**
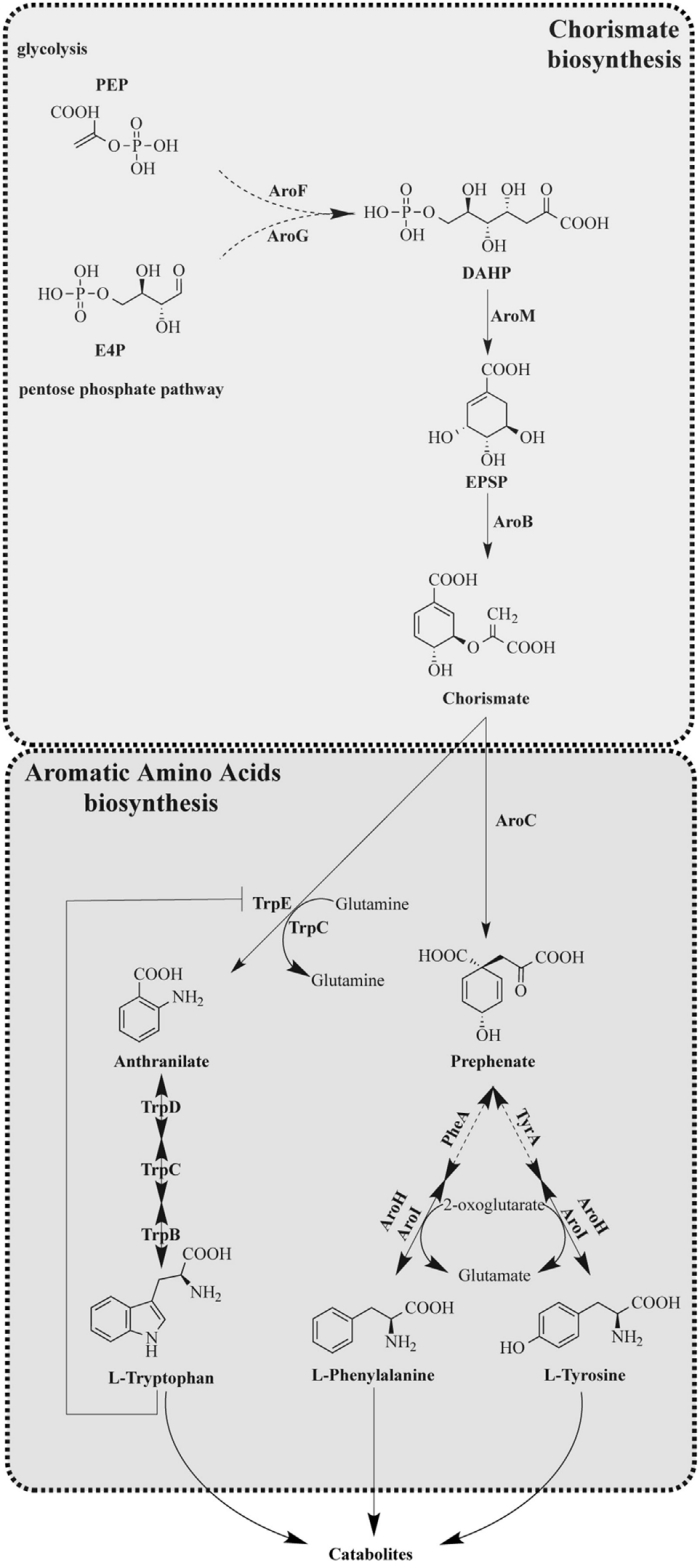
Tryptophan anabolism of *Aspergillus fumigatus*. [Modified from that of Wang et al. (28)]. Solid arrows indicate characterized reaction as being present in *A. fumigatus* with product detected. Dashed arrows indicate uncharacterized reactions; however, putative orthologs are present in *A. fumigatus*.

Chorismic acid derived from the shikimic acid pathway is a key intermediate in producing Trp, phenylalanine (Phe), and tyrosine (Tyr) in microorganisms including *A. fumigatus* (Figure 1). Trp and Phe are classified as essential amino acids, whereas mammals acquire them from diet, whereas Tyr is synthesized *via* the hydroxylation of Phe (23, 24). The absence of the AAA biosynthetic enzymes and the low bioavailability of Trp in humans makes the Trp biosynthetic enzymes attractive targets for antifungals (25).

### Fungal Trp Anabolic Pathway

Aromatic amino acid synthesis has been extensively studied in *Saccharomyces cerevisiae* and provides the basis for the functional characterization of orthologous enzymes in filamentous fungi (23, 24, 26–28). The shikimic acid pathway is a 7-enzymatic step reaction that initiates with two substrates, phosphoenolpyruvate (PEP) and erythrose-4-phosphate (E4P), which are intermediates of glycolysis and pentose phosphate pathways, respectively (29) (Figure 1). The first step of the shikimic acid pathway is catalyzed by 3-deoxy-d-arabinoheptulosonate 7-phosphate (DAHP) synthase to convert PEP and E4P to DAHP. In *S. cerevisiae* and *A. nidulans*, there are two DAHP synthases, Aro3 and Aro4, which are allosterically inhibited by phenylalanine and tyrosine, respectively (24). Steps 2–6 in filamentous fungi such as *A. nidulans* and *A. fumigatus* are completed by the pentafunctional enzyme AroM, or Aro1 in the model organism *S. cerevisiae* (30). The shikimate pathway culminates in the production of chorismic acid synthesized by the enzyme chorismate synthase (AroB) from 5-enolpyruvylshikimate-3-phosphate (EPSP) (31).

The synthesis of Trp from chorismate is initiated by an anthranilate synthase (AS), which converts chorismate to anthranilate, followed by three enzymatic steps as presented in Figure 1 with the respective functions outlined in Table 2. AS(s) in *S. cerevisiae* have been characterized, and it consists of two subunits: anthranilate synthase subunit I (AAS-I), which binds chorismate and is subject to feedback inhibition by Trp and anthranilate synthase subunit II (AAS-II) which is a glutamine amidotransferase (32). The *A. nidulans trpC*, an AAS-II encoding gene, was characterized in 1977 (33) and found exchangeable with an *A. fumigatus trpC* in 1994 (34). Wang et al. (28) recently characterized *trpE*, the AAS-I encoding gene in *A. fumigatus*. Wang et al. (28) explored the functions of two putative AAS-Is termed *trpE* and *icsA* by creating null mutants. The deletion of *trpE* led to a Trp auxotrophic strain, whereas the deletion of *icsA* did not. To ensure that *icsA* did not serve a redundant role for Trp synthesis, the group overexpressed *icsA* in a *trpE* deletion and showed that the overexpression of *icsA* does not reverse the Trp auxotrophy concluding that TrpE is the only AS in *A. fumigatus*. Interestingly, the group showed that IcsA is an active enzyme in *A. fumigatus* as the precursor-chorismate pool is altered in the absence or overproduction of IcsA; however, the product is not known (28). Sasse et al. also confirmed that deletion of *trpE* (they termed *trpA*) results in an *A. fumigatus* Trp auxotrophy (31).

**Table 2:** *Aspergillus fumigatus* tryptophan (Trp) metabolism genes and putative protein function.

Protein	Ortholog in *Saccharomyces cerevisiae*	Gene name in *A. fumigatus*	Protein function^a^	Ortholog in mammals
**Chorismate biosynthesis**				
AroF	Aro3	Afu1g02110	DAHP synthase	–
AroG	Aro4	Afu7g04070		
AroM	Aro1	Afu1g13740	EPSP synthase	–
AroB	Aro2	Afu1g06940	Chorismate synthase	–

**Aromatic amino acid (AAA) biosynthesis**				

TrpE	Trp2	Afu6g12580	Anthranilate synthase	–
TrpC	Trp3	Afu1g13090		
TrpD	Trp4	Afu4g11980	Anthranilate phosphoribosyltransferase	–
TrpC	Trp1	Afu1g13090	Phosphoribosylanthranilate isomerase	–
TrpB	Trp5	Afu2g13250	Trp synthase	–
IcsA	–	Afu6g12110	Isochorismate synthase	–
AroC	Aro7	Afu5g13130	Chorismate mutase	–
PheA	Pha2	Afu5g05690	Prephenate dehydratase	–
TyrA	Tyr1	Afu2g10450	Prephenate dehydrogenase	–
AroH	Aro8	Afu2g13630	AAA transaminase	–
AroI	Aro9	Afu5g02290		

**Trp degradation**				

IdoA	Bna2	Afu3g14250	Indoleamine 2,3-dioxygenases	IDO1
IdoB	Bna2	Afu4g09830		IDO2
IdoC	Bna2	Afu7g02010		TDO
FmdS	Bna7	Afu1g09960	Kynurenine formamidase	AFMID
Bna4	Bna4	Afu6g07340	Kynureninase	
Bna5	Bna5	Afu4g09840		KYNU
AroH	Aro8	Afu2g13630	AAA transaminase	LAAO
AroI	Aro9	Afu5g02290		
AadA	–	Afu3g02240	Trp carboxylase	AADC
MaoN	–	Afu3g00100	Monoamine oxidase	MAOA
AldA	Ald4	Afu2g00720	Aldehyde dehydrogenase	ALDH
	Ald5	Afu7g01000		
		Afu6g11430		

*^a^Prediction of protein function based on AspGD (http://www.aspgd.org/) and KEGG (http://www.genome.jp/kegg/kegg2.html)*.

*DAHP synthase, 3-deoxy-d-arabinoheptulosonate 7-phosphate synthase; EPSP synthase, enolpyruvylshikimate-3-phosphate synthase; AFMID, arylformamidase; KYNU, kynureninase; LAAO, l-amino-acid oxidase; AADC, aromatic-l-amino-acid decarboxylase, MAOA, monoamine oxidase; ALDH2, aldehyde dehydrogenase family*.

### The Shikimate Pathway As Potential Antifungal Targets

Currently, there are four major classes of antifungals: azoles and amphotericin B targeting ergosterol, 5-fluorocytosine targeting DNA synthesis, and echinocandins targeting cell wall synthesis. These antifungals either exhibit high toxicity to the mammalian cell (particularly amphotericin B and 5-fluorocytosine) or lose efficacy due to the emergence of drug resistant strains (azoles and echinocandins) (9). With *A. fumigatus* being a eukaryotic pathogen and sharing many proteins with mammalian hosts, there are limitations to developing effective and safe antifungals and therefore a great need for treatments that are fungal specific. Since Trp is a human-essential amino acid and the enzymes in the biosynthesis are fungal specific, several studies have suggested utilizing and finding drugs to target the enzymes of this pathway (35–38).

Targeting essential amino acid pathways have already shown potential for new classes of antifungals. Several groups have explored inhibitors of genes or enzymes involved in methionine biosynthesis. Azoxybacillin, a compound isolated from *B. cereus* targets methionine biosynthesis by interfering with expression of homoserine transacetylase and sulfite reductase encoding genes (39–41). Whereas azoxybacillin displayed a broad spectrum antifungal activity *in vitro, in vivo* activity was low possibly due to bioavailability in the host (41). R1-331, a natural product from *Streptomyces akiyoshiensis*, is an effective inhibitor of homoserine dehydrogenase involved in both methionine and threonine biosynthesis (42, 43). Yamaguchi et al. show that R1-331 was active against medically important fungi such as *Candida albicans* and *Cryptoccocus neoformans* and proved to be effective in the treatment of systemic murine candidiasis (42, 44).

Compounds targeting AAA pathways are limited with the most famous being the herbicide Roundup, where the active ingredient glyphosate inhibits EPSP synthase, one of the first enzymes initiating the shikimate pathway (45) (Figure 1). Glyphosate has shown to inhibit growth of several fungi including *Candida maltose* (46), *Pneumocystis* (47), and *Cryptococcus neoformans* where glyphosate delayed fungal melanization *in vitro* and *in vivo* and prolonged mice survival during infection (48). Another inhibitor of AAA pathway is a fluorinated anthranilate moiety, 6-FABA, which targets the TrpE enzyme and showed bactericidal activity when used on *Mycobacterium tuberculosis* (49). The studies of these inhibitors suggest that the Trp biosynthetic pathway could be fruitful in future antifungal drug design.

The value of AAA pathways as drug targets is supported by the findings that AAA auxotrophic mutants are less virulent in animal infection models. Sasse et al. explored the possibility of these pathways as potential drug target by testing the virulence of several AAA auxotrophic mutants in a murine IPA model (31). This study demonstrated that AroM (Figure 1) was required for *A. fumigatus* viability. The group also constructed a conditional AroB repression strain that was attenuated virulence. Both a Trp auxotroph (TrpE mutant) and Tyr/Phe auxotroph (AroC mutant) were severely attenuated in virulence for pulmonary infection. Interestingly, the group also unveiled a putative difference in AAA distribution within the host by conducting a systemic infection showing that in a bloodstream infection the TrpE and the AroC mutants although less virulent, can establish some infection (31). Taken together, these results suggest that inhibitors of AAA biosynthetic pathways can potentially be used against *A. fumigatus* as a standalone treatment in a localized pulmonary infection or as an additive treatment in a systemic infection. The result of the bloodstream infection observed by Sasse et al. also suggests that there are mechanisms for the fungus to sense Trp in its environment and utilize it. In *S. cerevisiae*, the Trp specific permease, Tat2, is required for Trp uptake in yeast (50) and its closest homolog in *A. fumigatus* (Afu7g04290) is upregulated during fungal encounters with neutrophils (51) and dendritic cells (52). In *A. nidulans*, the G-protein coupled receptor (Gpr) H may be responsible for sensing Trp and glucose and GprH is conserved in *A. fumigatus* (53, 54). Perhaps, for a systemic infection, inhibition of specific permeases or development of a GprH antagonist would be useful in reducing infections by *Aspergillus*.

## Catabolic Trp Metabolites in Fungi and Host

**Figure 2:**
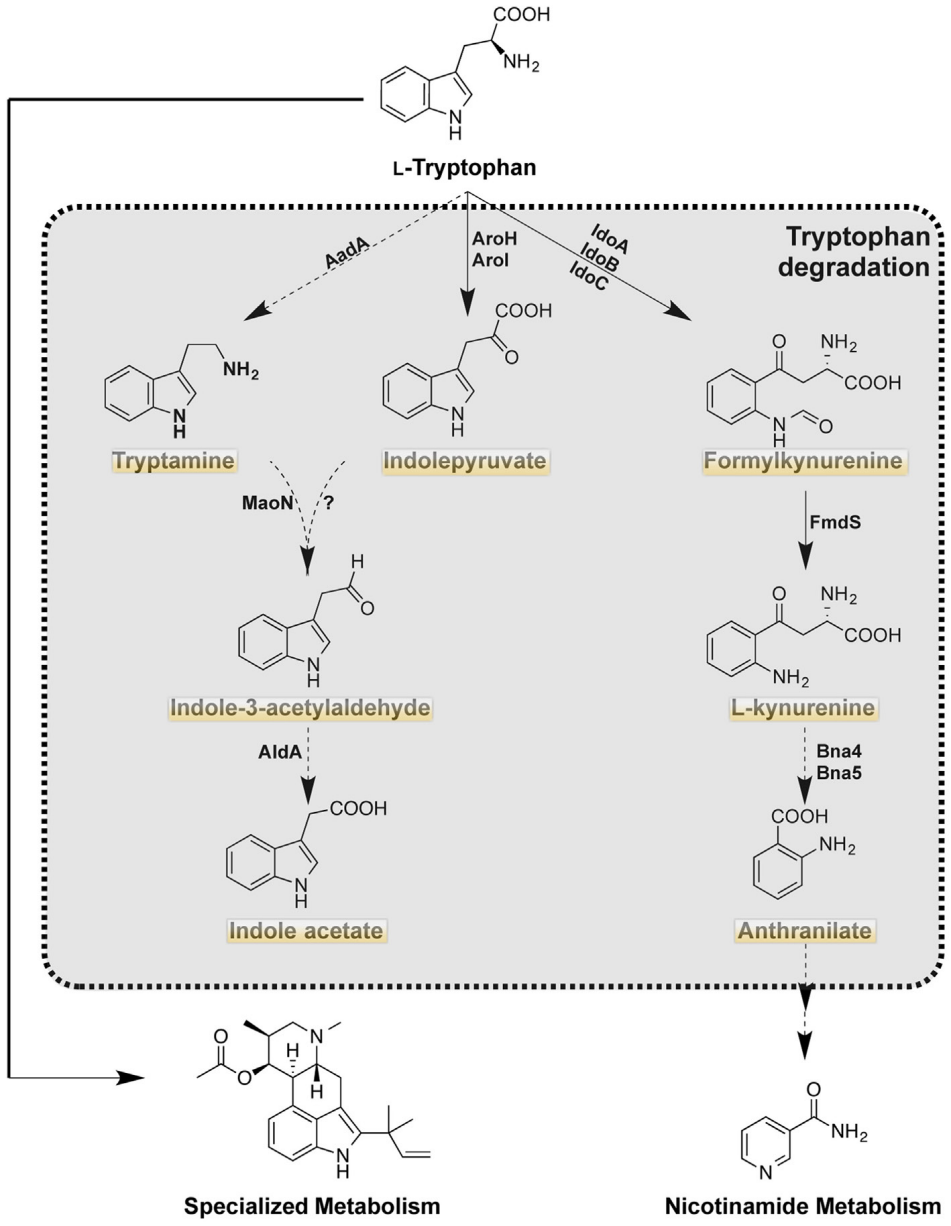
Tryptophan catabolism of *Aspergillus fumigatus*. Highlighted products are putatively produced in the mammalian host as the orthologous enzymes are present in the host (http://www.genome.jp/kegg). Solid arrows indicate characterized reaction as being present in *A. fumigatus* with product detected. Dashed arrows indicate uncharacterized reactions, however putative orthologs are present in *A. fumigatus*.

Although the anabolic Trp pathway is absent in mammals, the common catabolic pathways exist in the mammalian host with possession of the same enzymes as *A. fumigatus* (Figure 2). Through the expression of Trp degradation enzymes, immune cells are both controlling inflammation and combating microbial infection. In addition to Trp degradation pathways conserved with animals, *A. fumigatus* can also direct Trp pools to secondary metabolites that may impact host health and response (Table 1).

### Fungal Trp Catabolism

There are three putative pathways (Figure 2; Table 2) for the degradation of Trp in *A. fumigatus*. The kynurenine branch is catalyzed by IDOs that convert Trp into formylkynurenine. In *A. fumigatus*, there are 3 putative *ido* genes: *idoA, idoB*, and *idoC*, the orthologs of *A. oryzae idoα, idoβ*, and *ido*γ, respectively (55). Enzymatic studies of *Aspergillus oryzae* IDO enzymes suggest two of the three enzymes, IDO*α*, and IDO*β*, may participate in Trp degradation as they have a higher affinity of its substrate. However the recent study by Wang et al., suggested IDOb might be the more dominant enzyme than IDOa as determined by gene expression of *A. fumigatus* grown on Trp amended media (28). Additionally, *idoC* gene expression was slightly induced by the addition of Trp, but the authors note that IDOc had a closer relationship to bacterial IDOs than to fungal IDOs (28, 55). In *S. cerevisiae*, formylkynurenine is further oxidized to the immunomodulatory product kynurenine by a kynurenine formamidase denoted as Bna7, which has been described in *A. nidulans* and is predicted to be involved in NAD (+), biosynthesis (56). The kynurenine branch in fungi is involved in the *de novo* biosynthesis of NAD (+), a coenzyme that is required for oxidation-reduction reactions (57).

Tryptophan can also be metabolized via the indole pyruvate pathway, initiated through the transamination of Trp by aromatic aminotransferases (termed Aro8 and Aro9 in *S. cerevisiae*). These aromatic aminotransferases are also involved in the synthesis of Phe and Tyr in *S. cerevisiae*, and their orthologs are present in *A. fumigatus* (28). In *S. cerevisiae*, the deletion of both Aro8 and Aro9 results in Phe and Tyr auxotrophies (24, 58, 59). In *Candida* spp., where filamentation and pigment production play a role in virulence, the products of these enzymes have been described to influence both phenotypes. The deletion of *aro*8 in *C. glabrata* results in a reduced pigment production and leads to an increased sensitivity to hydrogen peroxide (27). The *aro*8 and *aro*9 mutants of *C. albicans* results in a decreased conversion of Trp to indole acetaldehyde, which is formed via decarboxylation of indole pyruvate. Filamentation of *C. albicans* increased with the exposure to indole acetaldehyde (60).

Indole acetaldehyde can also be produced via the third putative product of Trp degradation: tryptamine. Tryptamine is most famously known as the active compound in psilocybin and for its similarity to serotonins (61). Although the production of tryptamine has yet to be described in *A. fumigatus*, the downstream product of the tryptamine and indole pyruvate pathway, indole acetic acid has been described in several *Aspergillus* spp. including *A. fumigatus* (62, 63). Downstream metabolism of kynurenine, indole pyruvate, and tryptamine has not been explored further, but *A. fumigatus* does possess putative enzymes for the re-synthesis of anthranilate, the precursor to Trp (as denoted in Figures 1 and 2).

### AAA Incorporation into *Aspergillus* Toxins

Many filamentous fungi, including *A. fumigatus*, produce bioactive small molecules that can have detrimental impacts on human health. Subsets of these toxins are small peptides synthesized by non-ribosomal peptide synthetases (NRPS). Several pathogenic *Aspergillus* species synthesize AAA derived peptides including gliotoxin (*Phe* and serine) (64), fumiquinazoline (*Trp, Anthranilate*, and Alanine) (13), fumigaclavine (*Trp*) (65), fumitremorgin (*Trp* and Proline) (66), hexadehydroastechrome (*Trp* and Alanine) (21), fumisoquin (*Tyr*, Serine, and Methionine) (14), DPP-IV inhibitor WYK-1 (*Trp, Tyr*, and Leucine) (67), cyclopiazonic acid (*Trp*) (68) and benzomalvin (*Phe* and *Anthranilate*) (69). Table 1 summarizes the known AAA derived secondary metabolites of *A. fumigatus* and their effect on the host.

Although, fumiquinazolines have yet to be assessed for virulence in an animal model, they are known to have cytotoxic properties (13). Fumigaclavines have been described to have immunosuppressive properties in several studies including the suppression of antifungal cytokines such as TNFα, IL-17, and IFN-γ (12). Fumitremorgin, verruculogen, and tryprostatin—all related products of the fumitremorgin pathway—induce tremorgenic activity in mice and act on the central nervous system (15, 66, 70, 71). Mutants in the hexadehydroastechrome pathway (21) and gliotoxin (20, 21) pathways have altered virulence in murine IPA models. Decreased virulence of the *gliP* mutant (GliP is the NRPS required for gliotoxin synthesis) is dependent on host immune status [reviewed in Ref. (7)]. Overexpression of *hasA* encoding the hexadehydroastechrome transcription factor and thus leading to increased hexadehydroastechrome production was more virulent than wild type *A. fumigatus* in a neutropenic model of IPA (21). Although the exact mechanism underlying the increased virulence of the OE::*hasA* strain is unknown, iron homeostasis and cross talk between metabolic pathways may contribute to the increased virulence of OE::*hasA* (22). These studies highlight the potential contribution of AAA derived toxins in virulence of *A. fumigatus*.

### Host Trp Catabolism *via* IDO

The function of host IDO during mammalian infection was originally thought to center on the anti-proliferative effects of pathogenic microorganism via deprivation of Trp exerted by the host. IDO is up-regulated by interferon gamma (IFNγ) and depletes Trp (the least abundant essential amino acid) to inhibit pathogen expansion (72, 73), as demonstrated in the constraint of chlamydial growth (74). Numerous studies have since implicated IDO activity as important in fungal infections and have reported the relative outcomes of IDO expression on disease progression (Table 3). Accumulating data continues to support that IDO participates in the host–pathogen interaction in human epithelial cells; therefore, the co-evolution of host and microbe Trp metabolism has been investigated (75). The current consensus is that IDO activation is pivotal in regulating inflammatory processes directly via Trp depletion and indirectly via the IDO-mediated release of Trp catabolic secondary metabolites (namely, kynurenines).

**Table 3:** Summary of studies where IDO enzyme activity was found to be implicated in fungal infections.

Fungus	Mouse model	IDO	Outcome	Reference
*Candida albicans*	Gastrointestinal inf.	Upregulation	Protection of the host against fungus	(76)
*C. albicans*	Gastrointestinal inf.	Upregulation	Protection of the host against fungus	(77)
*C. albicans*	Gastrointestinal inf.	Upregulation	Protection of the host against fungus	(78)
*C. albicans*	*In vitro*	Downregulation	Not done	(79)
*Aspergillus fumigatus*	Keratitis	Upregulation	Protection of the host against fungus	(80)
*A. fumigatus*	Allergy	Overexpression	Protection of the host against fungus	(81)
*A. fumigatus*	IPA	Upregulation	Protection of the host against fungus	(82)
*A. fumigatus*	IPA in chronic granulomatous disease mice	Upregulation	Protection of the host against fungus	(83)
*A. fumigatus*	IPA in CF mice	Upregulation	Protection of the host against fungus	(84)
*Paracoccidioides brasiliensis*	Pulmonary infection	Upregulation	Protection of the host against fungus	(85)
*P. brasiliensis*	Pulmonary infection	Upregulation	Protection of the host against fungus	(86)
*P. brasiliensis*	Pulmonary infection	Upregulation	Protection of the host against fungus	(87)
*Histoplasma capsulatum*	Pulmonary infection	Downregulation	Protection of the host against fungus	(88)
*H. capsulatum*	Pulmonary infection	Upregulation	Protection of the host against fungus	(89)

Dietary Trp is catabolized by two different IDO protein isoforms, IDO1 and IDO2 that are expressed by immune cells, and TDO (Trp 2,3-dioxygenase) that is mainly expressed in the liver. Cells involved in the innate processes of the anti-microbial defense, such as dendritic cells (DCs), neutrophils, and macrophages express IDO1 upon microbial encounter mainly *via* toll-like receptor stimulation. How fungi specifically induce IDO expression is not known; however, induction by other pathogens is associated with pathogen associated molecular patterns, including lipopolysaccharides and CpG oligodeoxynucleotides (78, 90–92), underlining a role for kynurenine metabolism in microbial-induced inflammatory processes.

### IDO-Mediated Tolerance: Impacts on Antimicrobial Responses

Evolutionary studies have shown that the host immune defense against microbes is characterized by three different mechanisms: avoidance, resistance, and tolerance (93). Modules of immunity provide resistance to limit pathogen burden and tolerance and host damage caused by the immune reaction *per se*. However, the inflammatory reaction, although largely considered beneficial for its antimicrobial functions, may also contribute to pathogenicity. Thus, rescue from infection pathology may not only depend on microbial colonization (and inactivation of resistance mechanisms) but also on the resolution of tissue inflammatory pathology through tolerogenic responses to pathogens (94).

Studies using a mouse model of mucosal or invasive *C. albicans* infection found that systemic inhibition of IDO *in vivo* reduced gastrointestinal inflammation and unexpectedly, elevated the levels of fungal colonization compared to control mice (77). Notably, tolerogenic responses toward *C. albicans* were abrogated when IDO was antagonized *in vivo*, as shown in various models of inflammatory disorders (95, 96). As with *C. albicans*, IDO and kynurenine production during *A. fumigatus* infection contributes to fungal pathogen eradication and the regulation of an unacceptable level of tissue damage (97). Indeed, IDO can increase kynurenine host levels to induce adaptive Treg expansion while limiting Th17 polarization (83, 96). In this context, the Th17 pathway, which downregulates Trp catabolism, may instead favor pathology and better explain the paradoxical correlation between fungal infection and chronic inflammation (98).

Another example of this paradox was demonstrated in the context of CGD, in which an NADPH oxidase defect results in reduced host production of antimicrobial ROS and extreme susceptibility to *Aspergillus* infections (1, 83). Although human studies have excluded a role for IDO in CGD (99), further investigations into the IDO pathway are warranted as such studies have failed to demonstrate functional IDO activity at sites of chronic inflammation. Measures of IDO functional activity during IPA have, however, been made in mouse models, and implicate defective IDO activity as a key mediator of chronic inflammation in CGD (83). An exaggerated Th17 pulmonary response was associated with reduced fungal clearance in mouse models of CGD that develop IPA. Here, reduced IDO function was directly related to NADPH/ROS deficiency, as ROS is essential for IDO catalytic activity in mammals (100). ROS deficiency as a result of reduced NADPH function, significantly enhanced IL-17 inflammation and fungal germination in the lung, thus further reducing neutrophil-mediated antimicrobial activities (83).

Since regulation of homeostasis and peripheral tolerance are extremely important in prevention of invasive Aspergillosis or allergy to *Aspergillus* antigens (97, 101), the role of IDO has been extensively studied in this model of fungal infection (81, 82, 102). These studies highlight the induction of the IDO metabolic pathway at different site of fungal colonization as keratinocytes or lung as well as the important anti-inflammatory activity of IDO in the tissue microenvironment (80–82, 84).

### Aryl Hydrocarbon Receptor (AhR) Activation by IDO Metabolites in Mammals: Biological Consequences

The AhR is a ligand-activated transcription factor first identified for its role during embryonic development and induction of xenobiotic metabolizing enzymes as a response to environmental toxins, such as dioxin (103). More recently, AhR has been shown to play a critical role in immunity by acting as an immune modulator during fungal infection (85). The connection between the AhR and the immune response lies in part in the endogenous Ahr ligands, which comprise many Trp metabolites, including kynurenine (104). Microbial Trp-derived metabolites can activate the AhR, leading to adjustments in the immune response that may hinder disease development (105). The AhR–IDO axis has been recently demonstrated in fungal infection, highlighting a role for IDO-derived metabolites to trigger AhR target genes (85, 106). For example, one AhR target gene, *Il22*, has been widely studied in the context of fungal/microbial infections (105, 107–109). AhR activation by IDO metabolites can also mediate the expansion of peripheral Treg with anti-inflammatory properties. Using IDO-deficient mice, increased pulmonary disease caused by *Paracoccidioides brasiliensis* was associated with decreased Treg expansion and reduced AhR protein expression (85). In murine models of IPA, distinct Treg populations capable of mediating anti-inflammatory effects expand following exposure to *Aspergillus* conidia (97). Late in infection, tolerogenic adaptive Treg (with shared phenotypic identity with the Treg controlling autoimmune diseases or diabetes) produce IL-10 and TGFβ, inhibit Th2 cells, and prevent an allergic reaction to *Aspergillus* (97).

## Conclusion

The interplay of Trp metabolic pathways and fungal/host interactions is intriguing with many unanswered questions of the exact nature of crosstalk of shared metabolites and consequences of activation of Trp degradative pathways. In *Aspergillus* infections in particular, not only does the pathogen synthesize and degrade Trp but it also can utilize this amino acid (and its precursor anthranilate or the other two AAAs Tyr and Phe) to yield several potentially damaging toxins (Table 1). Also, as both host and *Aspergillus* share catabolic IDO pathways, it is unclear which organism may generate immunomodulatory Trp degradation products and if they respond to each other’s products (e.g., kynurenine). Development of *A. fumigatus* IDO mutants for investigation of disease development could yield valuable information on this front. The research on the expression host IDOs exhibit the importance of an extremely coordinated immune response to mount the right inflammatory response for clearance of spores. However, while an increased IDO expression in the host can control inflammation, the suppression of the IDO-regulated antifungal Th17 responses can favor fungal growth. In this context, it will be critical to explore the entire IDO-mediated innate response, including the specific T cell regulatory subsets affected by IDO activity.

Although the Trp catabolic pathway is shared between host and pathogen, the anabolic pathway is unique to *A. fumigatus*. The antifungals currently used in treatment are becoming increasingly ineffective with emerging drug resistant strains; therefore, drugs targeting essential fungal specific pathways are needed. A proposal for fungal treatment has been highlighted through the studies of essential amino acids. As AAA mutants are auxotrophs and decreased in virulence (28, 31), investigations of drugs targeting these pathway enzymes could lead to novel antifungal compounds. Indeed, a few compounds have exhibited some efficacy in targeting Trp metabolic pathways in *M. tuberculosis* and several fungi and efforts to identify additional inhibitors are warranted.

## Author Contributions

TC, TZ, and NK have made a substantial, direct, and intellectual contribution to the work and LR provided intellectual insights to the revision. All authors have approved it for publication.

## Conflict of Interest Statement

The authors declare that the research was conducted in the absence of any commercial or financial relationships that could be construed as a potential conflict of interest.

## References

[1] Segal BH Romani LR. Invasive aspergillosis in chronic granulomatous disease. Med Mycol (2009) 47:S282–90. 10.1080/13693780902736620 19296367

[2] Keller NP Heterogeneity confounds establishment of “a” model microbial strain. MBio (2017) 8:e00135–17. 10.1128/mBio.00135-17 28223452 PMC5358909

[3] Krappmann S Bignell EM Reichard U Rogers T Haynes K Braus GH. The *Aspergillus fumigatus* transcriptional activator CpcA contributes significantly to the virulence of this fungal pathogen. Mol Microbiol (2004) 52:785–99. 10.1111/j.1365-2958.2004.04015.x 15101984

[4] Amich J Dumig M O’keeffe G Binder J Doyle S Beilhack A Exploration of sulfur assimilation of *Aspergillus fumigatus* reveals biosynthesis of sulfur-containing amino acids as a virulence determinant. Infect Immun (2016) 84:917–29. 10.1128/IAI.01124-15 26787716 PMC4807484

[5] D’Enfert C Diaquin M Delit A Wuscher N Debeaupuis JP Huerre M Attenuated virulence of uridine-uracil auxotrophs of *Aspergillus fumigatus*. Infect Immun (1996) 64:4401–5. 8926121 10.1128/iai.64.10.4401-4405.1996 PMC174389

[6] Rhodes JC. *Aspergillus fumigatus*: growth and virulence. Med Mycol (2006) 44(Suppl 1):S77–81. 10.1080/13693780600779419 17050423

[7] Dagenais TR Keller NP. Pathogenesis of *Aspergillus fumigatus* in invasive aspergillosis. Clin Microbiol Rev (2009) 22:447–65. 10.1128/CMR.00055-08 19597008 PMC2708386

[8] Sanglard D. Emerging threats in antifungal-resistant fungal pathogens. Front Med (2016) 3:11. 10.3389/fmed.2016.00011 27014694 PMC4791369

[9] Perlin DS Rautemaa-Richardson R Alastruey-Izquierdo A. The global problem of antifungal resistance: prevalence, mechanisms, and management. Lancet Infect Dis (2017) 17(12):e383–92. 10.1016/S1473-3099(17)30316-X 28774698

[10] Mackenzie CR Heseler K Muller A Daubener W. Role of indoleamine 2,3-dioxygenase in antimicrobial defence and immuno-regulation: tryptophan depletion versus production of toxic kynurenines. Curr Drug Metab (2007) 8:237–44. 10.2174/138920007780362518 17430112

[11] Zhu YX Yao LY Jiao RH Lu YH Tan RX. Enhanced production of fumigaclavine C in liquid culture of *Aspergillus fumigatus* under a two-stage process. Bioresour Technol (2014) 152:162–8. 10.1016/j.biortech.2013.10.089 24291794

[12] Guo W Hu S Elgehama A Shao F Ren R Liu W Fumigaclavine C ameliorates dextran sulfate sodium-induced murine experimental colitis via NLRP3 inflammasome inhibition. J Pharmacol Sci (2015) 129:101–6. 10.1016/j.jphs.2015.05.003 26320672

[13] Lim FY Ames B Walsh CT Keller NP. Co-ordination between BrlA regulation and secretion of the oxidoreductase FmqD directs selective accumulation of fumiquinazoline C to conidial tissues in *Aspergillus fumigatus*. Cell Microbiol (2014) 16:1267–83. 10.1111/cmi.12284 24612080 PMC4114987

[14] Baccile JA Spraker JE Le HH Brandenburger E Gomez C Bok JW Plant-like biosynthesis of isoquinoline alkaloids in *Aspergillus fumigatus*. Nat Chem Biol (2016) 12:419–24. 10.1038/nchembio.2061 27065235 PMC5049701

[15] Suzuki S Kikkawa K Yamazaki M. Abnormal behavioral effects elicited by a neurotropic mycotoxin, fumitremorgin A in mice. J Pharmacobiodyn (1984) 7:935–42. 10.1248/bpb1978.7.935 6152472

[16] Frisvad JC Larsen TO Extrolites of *Aspergillus fumigatus* and other pathogenic species in *Aspergillus* section fumigati. Front Microbiol (2015) 6:1485 10.3389/fmicb.2015.01485 26779142 PMC4703822

[17] Usui T Kondoh M Cui CB Mayumi T Osada H. Tryprostatin A, a specific and novel inhibitor of microtubule assembly. Biochem J (1998) 333(Pt 3):543–8. 10.1042/bj3330543 9677311 PMC1219615

[18] Khoufache K Puel O Loiseau N Delaforge M Rivollet D Coste A Verruculogen associated with *Aspergillus fumigatus* hyphae and conidia modifies the electrophysiological properties of human nasal epithelial cells. BMC Microbiol (2007) 7:5. 10.1186/1471-2180-7-5 17244350 PMC1797047

[19] Feng Y Holte D Zoller J Umemiya S Simke LR Baran PS. Total synthesis of verruculogen and fumitremorgin A enabled by ligand-controlled C-H borylation. J Am Chem Soc (2015) 137:10160–3. 10.1021/jacs.5b07154 26256033 PMC4777340

[20] Sugui JA Pardo J Chang YC Zarember KA Nardone G Galvez EM Gliotoxin is a virulence factor of *Aspergillus fumigatus*: *gliP* deletion attenuates virulence in mice immunosuppressed with hydrocortisone. Eukaryot Cell (2007) 6:1562–9. 10.1128/EC.00141-07 17601876 PMC2043361

[21] Yin WB Baccile JA Bok JW Chen Y Keller NP Schroeder FC. A nonribosomal peptide synthetase-derived iron(III) complex from the pathogenic fungus *Aspergillus fumigatus*. J Am Chem Soc (2013) 135:2064–7. 10.1021/ja311145n 23360537 PMC3590312

[22] Wiemann P Lechner BE Baccile JA Velk TA Yin WB Bok JW Perturbations in small molecule synthesis uncovers an iron-responsive secondary metabolite network in *Aspergillus fumigatus*. Front Microbiol (2014) 5:530. 10.3389/fmicb.2014.00530 25386169 PMC4208449

[23] Lingens F Regulation of aromatic amino acid biosynthesis in microorganisms. Acta Microbiol Acad Sci Hung (1976) 23:161–6. 9782

[24] Braus GH. Aromatic amino acid biosynthesis in the yeast *Saccharomyces cerevisiae*: a model system for the regulation of a eukaryotic biosynthetic pathway. Microbiol Rev (1991) 55:349–70. 1943992 10.1128/mr.55.3.349-370.1991 PMC372824

[25] Tagliamonte A Gessa R Biggio G Vargiu L Gessa GL Daily changes of free serum tryptophan in humans. Life Sci (1974) 14:349–54. 10.1016/0024-3205(74)90065-4 4813595

[26] Pereira SA Livi GP. Aromatic amino-acid biosynthesis in *Candida albicans*: identification of the ARO4 gene encoding a second DAHP synthase. Curr Genet (1996) 29:441–5. 10.1007/BF02221512 8625423

[27] Brunke S Seider K Almeida RS Heyken A Fleck CB Brock M *Candida glabrata* tryptophan-based pigment production via the Ehrlich pathway. Mol Microbiol (2010) 76:25–47. 10.1111/j.1365-2958.2010.07052.x 20199593

[28] Wang PM Choera T Wiemann P Pisithkul T Amador-Noguez D Keller NP. TrpE feedback mutants reveal roadblocks and conduits toward increasing secondary metabolism in *Aspergillus fumigatus*. Fungal Genet Biol (2016) 89:102–13. 10.1016/j.fgb.2015.12.002 26701311 PMC4789178

[29] Hawkins AR Lamb HK Moore JD Charles IG Roberts CF The pre-chorismate (shikimate) and quinate pathways in filamentous fungi: theoretical and practical aspects. J Gen Microbiol (1993) 139:2891–9. 10.1099/00221287-139-12-2891 8126417

[30] Duncan K Edwards RM Coggins JR. The pentafunctional AroM enzyme of *Saccharomyces cerevisiae* is a mosaic of monofunctional domains. Biochem J (1987) 246:375–86. 10.1042/bj2460375 2825635 PMC1148286

[31] Sasse A Hamer SN Amich J Binder J Krappmann S. Mutant characterization and *in vivo* conditional repression identify aromatic amino acid biosynthesis to be essential for *Aspergillus fumigatus* virulence. Virulence (2016) 7:56–62. 10.1080/21505594.2015.1109766 26605426 PMC4871646

[32] Graf R Mehmann B Braus GH. Analysis of feedback-resistant anthranilate synthases from *Saccharomyces cerevisiae*. J Bacteriol (1993) 175:1061–8. 10.1128/jb.175.4.1061-1068.1993 8432699 PMC193020

[33] Kafer E The anthranilate synthetase enzyme complex and the trifunctional *trpC* gene of *Aspergillus*. Can J Genet Cytol (1977) 19:723–38. 10.1139/g77-079 610841

[34] Borgia PT Dodge CL Eagleton LE Adams TH. Bidirectional gene transfer between *Aspergillus fumigatus* and *Aspergillus nidulans*. FEMS Microbiol Lett (1994) 122:227–31. 10.1111/j.1574-6968.1994.tb07172.x 7988865

[35] Hu W Sillaots S Lemieux S Davison J Kauffman S Breton A Essential gene identification and drug target prioritization in *Aspergillus fumigatus*. PLoS Pathog (2007) 3:e24. 10.1371/journal.ppat.0030024 17352532 PMC1817658

[36] Blanco B Prado V Lence E Otero JM Garcia-Doval C Van Raaij MJ *Mycobacterium tuberculosis* shikimate kinase inhibitors: design and simulation studies of the catalytic turnover. J Am Chem Soc (2013) 135:12366–76. 10.1021/ja405853p 23889343

[37] Ianiri G Idnurm A. Essential gene discovery in the basidiomycete *Cryptococcus neoformans* for antifungal drug target prioritization. MBio (2015) 6:e02334–14. 10.1128/mBio.02334-14 25827419 PMC4453551

[38] Kaltdorf M Srivastava M Gupta SK Liang C Binder J Dietl AM Systematic identification of anti-fungal drug targets by a metabolic network approach. Front Mol Biosci (2016) 3:22. 10.3389/fmolb.2016.00022 27379244 PMC4911368

[39] Aoki Y Kondoh M Nakamura M Fujii T Yamazaki T Shimada H A new methionine antagonist that has antifungal activity: mode of action. J Antibiot (Tokyo) (1994) 47:909–16. 10.7164/antibiotics.47.909 7928678

[40] Aoki Y Kamiyama T Fujii T Yamamoto M Ohwada J Arisawa M. Design of an antifungal methionine inhibitor not antagonized by methionine. Biol Pharm Bull (1995) 18:1267–71. 10.1248/bpb.18.1267 8845820

[41] Aoki Y Yamamoto M Hosseini-Mazinani SM Koshikawa N Sugimoto K Arisawa M. Antifungal azoxybacilin exhibits activity by inhibiting gene expression of sulfite reductase. Antimicrob Agents Chemother (1996) 40:127–32. 8787893 10.1128/aac.40.1.127 PMC163070

[42] Yamaguchi M Yamaki H Shinoda T Tago Y Suzuki H Nishimura T The mode of antifungal action of (S)2-amino-4-oxo-5-hydroxypentanoic acid, RI-331. J Antibiot (Tokyo) (1990) 43:411–6. 10.7164/antibiotics.43.411 2190964

[43] Jacques SL Ejim LJ Wright GD. Homoserine dehydrogenase from *Saccharomyces cerevisiae*: kinetic mechanism and stereochemistry of hydride transfer. Biochim Biophys Acta (2001) 1544:42–54. 10.1016/S0167-4838(00)00202-8 11341915

[44] Yamaki H Yamaguchi M Nishimura T Shinoda T Yamaguchi H. Unique mechanism of action of an antifungal antibiotic RI-331. Drugs Exp Clin Res (1988) 14:467–72. 3071450

[45] Tan S Evans R Singh B. Herbicidal inhibitors of amino acid biosynthesis and herbicide-tolerant crops. Amino Acids (2006) 30:195–204. 10.1007/s00726-005-0254-1 16547651

[46] Bode R Melo C Birnbaum D. Mode of action of glyphosate in *Candida maltosa*. Arch Microbiol (1984) 140:83–5. 10.1007/BF00409776 6152388

[47] Chin K Wyder MA Kaneshiro ES Glyphosate reduces organism viability and inhibits growth *in vitro* of *Pneumocystis*. J Eukaryot Microbiol (1999) 46:139S–41S. 10519291

[48] Fernandes JD Martho K Tofik V Vallim MA Pascon RC The role of amino acid permease and tryptophan biosynthesis in *Cryptococcus neoformans* survival. PLoS One (2015) 10:e0132369 10.1371/journal.pone.0132369 26162077 PMC4498599

[49] Abdel-Rahman HM El-Koussi NA Hassan HY. Fluorinated 1,2,4-triazolo[1,5-a]pyrimidine-6-carboxylic acid derivatives as antimycobacterial agents. Arch Pharm (Weinheim) (2009) 342:94–9. 10.1002/ardp.200800113 19173243

[50] Forsberg H Gilstring CF Zargari A Martinez P Ljungdahl PO. The role of the yeast plasma membrane SPS nutrient sensor in the metabolic response to extracellular amino acids. Mol Microbiol (2001) 42:215–28. 10.1046/j.1365-2958.2001.02627.x 11679080

[51] Sugui JA Kim HS Zarember KA Chang YC Gallin JI Nierman WC Genes differentially expressed in conidia and hyphae of *Aspergillus fumigatus* upon exposure to human neutrophils. PLoS One (2008) 3:e2655. 10.1371/journal.pone.0002655 18648542 PMC2481287

[52] Morton CO Varga JJ Hornbach A Mezger M Sennefelder H Kneitz S The temporal dynamics of differential gene expression in *Aspergillus fumigatus* interacting with human immature dendritic cells *in vitro*. PLoS One (2011) 6:e16016. 10.1371/journal.pone.0016016 21264256 PMC3021540

[53] Grice CM Bertuzzi M Bignell EM. Receptor-mediated signaling in *Aspergillus fumigatus*. Front Microbiol (2013) 4:26. 10.3389/fmicb.2013.00026 23430083 PMC3576715

[54] Brown NA Dos Reis TF Ries LN Caldana C Mah JH Yu JH G-protein coupled receptor-mediated nutrient sensing and developmental control in *Aspergillus nidulans*. Mol Microbiol (2015) 98:420–39. 10.1111/mmi.13135 26179439

[55] Yuasa HJ Ball HJ. The evolution of three types of indoleamine 2,3 dioxygenases in fungi with distinct molecular and biochemical characteristics. Gene (2012) 504:64–74. 10.1016/j.gene.2012.04.082 22564706

[56] Fraser JA Davis MA Hynes MJ. The formamidase gene of *Aspergillus nidulans*: regulation by nitrogen metabolite repression and transcriptional interference by an overlapping upstream gene. Genetics (2001) 157:119–31. 11139496 10.1093/genetics/157.1.119 PMC1461490

[57] Ohashi K Kawai S Murata K. Secretion of quinolinic acid, an intermediate in the kynurenine pathway, for utilization in NAD+ biosynthesis in the yeast *Saccharomyces cerevisiae*. Eukaryot Cell (2013) 12:648–53. 10.1128/EC.00339-12 23457190 PMC3647768

[58] Urrestarazu A Vissers S Iraqui I Grenson M. Phenylalanine- and tyrosine-auxotrophic mutants of *Saccharomyces cerevisiae* impaired in transamination. Mol Gen Genet (1998) 257:230–7. 10.1007/s004380050643 9491082

[59] Bulfer SL Brunzelle JS Trievel RC Crystal structure of *Saccharomyces cerevisiae* Aro8, a putative alpha-aminoadipate aminotransferase. Protein Sci (2013) 22:1417–24. 10.1002/pro.2315 23893908 PMC3795499

[60] Rao RP Hunter A Kashpur O Normanly J. Aberrant synthesis of indole-3-acetic acid in *Saccharomyces cerevisiae* triggers morphogenic transition, a virulence trait of pathogenic fungi. Genetics (2010) 185:211–20. 10.1534/genetics.109.112854 20233857 PMC2870956

[61] Tylš F Páleníček T Horáček J Psilocybin – summary of knowledge and new perspectives. Eur Neuropsychopharmacol (2014) 24:342–56. 10.1016/j.euroneuro.2013.12.006 24444771

[62] Bilkay IS Karakoç S Aksoz N Indole-3-acetic acid and gibberellic acid production in *Aspergillus niger*. Turk J Biol (2010) 34:313–8. 10.3906/biy-0812-15

[63] Pattaeva M Bakhtiyor R Growth and phytohormones production by thermophilic *Aspergillus fumigatus* 2 and thermotolerant *Aspergillus terreus* 8 strains in salt stress. Br J Appl Sci Technol (2015) 8:305–12. 10.9734/BJAST/2015/12292

[64] Macdonald JC Slater GP. Biosynthesis of gliotoxin and mycelianamide. Can J Biochem (1975) 53:475–8. 10.1139/o75-066 1125828

[65] Mulinti P Allen NA Coyle CM Gravelat FN Sheppard DC Panaccione DG. Accumulation of ergot alkaloids during conidiophore development in *Aspergillus fumigatus*. Curr Microbiol (2014) 68:1–5. 10.1007/s00284-013-0434-2 23925951

[66] Yamazaki M Suzuki S Toxicology of tremorgenic mycotoxins, fumitremorgin A and B. Dev Toxicol Environ Sci (1986) 12:273–82. 2881766

[67] Imamura K Tsuyama Y Hirata T Shiraishi S Sakamoto K Yamada O Identification of a gene involved in the synthesis of a dipeptidyl peptidase IV inhibitor in *Aspergillus oryzae*. Appl Environ Microbiol (2012) 78:6996–7002. 10.1128/AEM.01770-12 22843525 PMC3457513

[68] Liu X Walsh CT. Cyclopiazonic acid biosynthesis in *Aspergillus* sp.: characterization of a reductase-like R* domain in cyclopiazonate synthetase that forms and releases cyclo-acetoacetyl-L-tryptophan. Biochemistry (2009) 48:8746–57. 10.1021/bi901123r 19663400 PMC2752376

[69] Clevenger KD Bok JW Ye R Miley GP Verdan MH Velk T A scalable platform to identify fungal secondary metabolites and their gene clusters. Nat Chem Biol (2017) 13:895–901. 10.1038/nchembio.2408 28604695 PMC5577364

[70] Yamazaki M Fujimoto H Kawasaki T Chemistry of tremorogenic metabolites. Fumitremorgin A from *Aspergillus fumigatus*. Chem Pharm Bull (Tokyo) (1980) 28:245–54. 10.1248/cpb.28.245 6988091

[71] Cole RJ Fungal tremorogens. Prikl Biokhim Mikrobiol (1993) 29:44–50. 8475020

[72] Colangelo EJ. Cervicocranium and the aviator’s protective helmet. Aviat Space Environ Med (1975) 46:1263–4. 1180788

[73] Zelante T Fallarino F Bistoni F Puccetti P Romani L Indoleamine 2,3-dioxygenase in infection: the paradox of an evasive strategy that benefits the host. Microbes Infect (2009) 11:133–41. 10.1016/j.micinf.2008.10.007 19007906

[74] Thomas SM Garrity LF Brandt CR Schobert CS Feng GS Taylor MW IFN-gamma-mediated antimicrobial response. Indoleamine 2,3-dioxygenase-deficient mutant host cells no longer inhibit intracellular *Chlamydia* spp. or *Toxoplasma* growth. J Immunol (1993) 150:5529–34. 8515074

[75] Aldajani WA Salazar F Sewell HF Knox A Ghaemmaghami AM. Expression and regulation of immune-modulatory enzyme indoleamine 2,3-dioxygenase (IDO) by human airway epithelial cells and its effect on T cell activation. Oncotarget (2016) 7:57606–17. 10.18632/oncotarget.11586 27613847 PMC5295376

[76] De Luca A Montagnoli C Zelante T Bonifazi P Bozza S Moretti S Functional yet balanced reactivity to *Candida albicans* requires TRIF, MyD88, and IDO-dependent inhibition of Rorc. J Immunol (2007) 179:5999–6008. 10.4049/jimmunol.179.9.5999 17947673

[77] Bozza S Fallarino F Pitzurra L Zelante T Montagnoli C Bellocchio S A crucial role for tryptophan catabolism at the host/*Candida albicans* interface. J Immunol (2005) 174:2910–8. 10.4049/jimmunol.174.5.2910 15728502

[78] Bonifazi P Zelante T D’angelo C De Luca A Moretti S Bozza S Balancing inflammation and tolerance *in vivo* through dendritic cells by the commensal *Candida albicans*. Mucosal Immunol (2009) 2:362–74. 10.1038/mi.2009.17 19421183

[79] Cheng SC Van De Veerdonk F Smeekens S Joosten LA Van Der Meer JW Kullberg BJ *Candida albicans* dampens host defense by downregulating IL-17 production. J Immunol (2010) 185:2450–7. 10.4049/jimmunol.1000756 20624941

[80] Jiang N Zhao G Lin J Hu L Che C Li C Indoleamine 2,3-dioxygenase is involved in the inflammation response of corneal epithelial cells to *Aspergillus fumigatus* infections. PLoS One (2015) 10:e0137423. 10.1371/journal.pone.0137423 26361229 PMC4567309

[81] Paveglio SA Allard J Foster Hodgkins SR Ather JL Bevelander M Campbell JM Airway epithelial indoleamine 2,3-dioxygenase inhibits CD4+ T cells during *Aspergillus fumigatus* antigen exposure. Am J Respir Cell Mol Biol (2011) 44:11–23. 10.1165/rcmb.2009-0167OC 20118221 PMC3028254

[82] De Luca A Bozza S Zelante T Zagarella S D’angelo C Perruccio K Non-hematopoietic cells contribute to protective tolerance to *Aspergillus fumigatus* via a TRIF pathway converging on IDO. Cell Mol Immunol (2010) 7:459–70. 10.1038/cmi.2010.43 20835271 PMC4002959

[83] Romani L Fallarino F De Luca A Montagnoli C D’angelo C Zelante T Defective tryptophan catabolism underlies inflammation in mouse chronic granulomatous disease. Nature (2008) 451:211–5. 10.1038/nature06471 18185592

[84] Iannitti RG Carvalho A Cunha C De Luca A Giovannini G Casagrande A Th17/Treg imbalance in murine cystic fibrosis is linked to indoleamine 2,3-dioxygenase deficiency but corrected by kynurenines. Am J Respir Crit Care Med (2013) 187:609–20. 10.1164/rccm.201207-1346OC 23306541

[85] Araujo EF Feriotti C Galdino NAL Preite NW Calich VLG Loures FV. The IDO-AhR axis controls Th17/Treg immunity in a pulmonary model of fungal infection. Front Immunol (2017) 8:880. 10.3389/fimmu.2017.00880 28791025 PMC5523665

[86] Araujo EF Medeiros DH Galdino NA Condino-Neto A Calich VL Loures FV. Tolerogenic plasmacytoid dendritic cells control *Paracoccidioides brasiliensis* infection by inducting regulatory T cells in an IDO-dependent manner. PLoS Pathog (2016) 12:e1006115. 10.1371/journal.ppat.1006115 27992577 PMC5215616

[87] Araujo EF Loures FV Bazan SB Feriotti C Pina A Schanoski AS Indoleamine 2,3-dioxygenase controls fungal loads and immunity in paracoccidioidomicosis but is more important to susceptible than resistant hosts. PLoS Negl Trop Dis (2014) 8:e3330. 10.1371/journal.pntd.0003330 25411790 PMC4238999

[88] Hage CA Horan DJ Durkin M Connolly P Desta Z Skaar TC *Histoplasma capsulatum* preferentially induces IDO in the lung. Med Mycol (2013) 51:270–9. 10.3109/13693786.2012.710857 23181600

[89] George MM Subramanian Vignesh K Landero Figueroa JA Caruso JA Deepe GS Jr. Zinc induces dendritic cell tolerogenic phenotype and skews regulatory T cell-Th17 balance. J Immunol (2016) 197:1864–76. 10.4049/jimmunol.1600410 27465530 PMC4992588

[90] Gorski JP Howard JB Effect of methylamine on the structure and function of the fourth component of human complement, C4. J Biol Chem (1980) 255:10025–8. 7430113

[91] Jung ID Lee C-M Jeong Y-I Lee JS Park WS Han J Differential regulation of indoleamine 2,3-dioxygenase by lipopolysaccharide and interferon gamma in murine bone marrow derived dendritic cells. FEBS Lett (2007) 581:1449–56. 10.1016/j.febslet.2007.02.073 17367785

[92] Fallarino F Volpi C Zelante T Vacca C Calvitti M Fioretti MC IDO mediates TLR9-driven protection from experimental autoimmune diabetes. J Immunol (2009) 183:6303–12. 10.4049/jimmunol.0901577 19841163

[93] Rivas FV Chervonsky AV Medzhitov R ART and immunology. Trends Immunol (2014) 35:451 10.1016/j.it.2014.09.002 25261059

[94] Soares MP Gozzelino R Weis S. Tissue damage control in disease tolerance. Trends Immunol (2014) 35:483–94. 10.1016/j.it.2014.08.001 25182198

[95] Romani L. Cell mediated immunity to fungi: a reassessment. Med Mycol (2008) 46:515–29. 10.1080/13693780801971450 19180748

[96] Zelante T Bozza S De Luca A D’angelo C Bonifazi P Moretti S Th17 cells in the setting of *Aspergillus* infection and pathology. Med Mycol (2009) 47(Suppl 1):S162–9. 10.1080/13693780802140766 18608926

[97] Montagnoli C Fallarino F Gaziano R Bozza S Bellocchio S Zelante T Immunity and tolerance to *Aspergillus* involve functionally distinct regulatory T cells and tryptophan catabolism. J Immunol (2006) 176:1712–23. 10.4049/jimmunol.176.3.1712 16424201

[98] De Luca A Pariano M Cellini B Costantini C Villella VR Jose SS The IL-17F/IL-17RC axis promotes respiratory allergy in the proximal airways. Cell Rep (2017) 20:1667–80. 10.1016/j.celrep.2017.07.063 28813677

[99] De Ravin SS Zarember KA Long-Priel D Chan KC Fox SD Gallin JI Tryptophan/kynurenine metabolism in human leukocytes is independent of superoxide and is fully maintained in chronic granulomatous disease. Blood (2010) 116:1755–60. 10.1182/blood-2009-07-233734 20511543 PMC2947395

[100] Macchiarulo A Camaioni E Nuti R Pellicciari R. Highlights at the gate of tryptophan catabolism: a review on the mechanisms of activation and regulation of indoleamine 2,3-dioxygenase (IDO), a novel target in cancer disease. Amino Acids (2009) 37:219–29. 10.1007/s00726-008-0137-3 18612775

[101] Zelante T De Luca A Bonifazi P Montagnoli C Bozza S Moretti S IL-23 and the Th17 pathway promote inflammation and impair antifungal immune resistance. Eur J Immunol (2007) 37:2695–706. 10.1002/eji.200737409 17899546

[102] Jiang N Zhao GQ Lin J Hu LT Che CY Li C Expression of indoleamine 2,3-dioxygenase in a murine model of *Aspergillus fumigatus* keratitis. Int J Ophthalmol (2016) 9:491–6. 10.18240/ijo.2016.04.03 27162718 PMC4853341

[103] Mackowiak B Wang H. Mechanisms of xenobiotic receptor activation: direct vs. indirect. Biochim Biophys Acta (2016) 1859:1130–40. 10.1016/j.bbagrm.2016.02.006 26877237 PMC4975672

[104] Hubbard TD Murray IA Perdew GH. Indole and tryptophan metabolism: endogenous and dietary routes to Ah receptor activation. Drug Metab Dispos (2015) 43:1522–35. 10.1124/dmd.115.064246 26041783 PMC4576673

[105] Zelante T Iannitti RG Cunha C De Luca A Giovannini G Pieraccini G Tryptophan catabolites from microbiota engage aryl hydrocarbon receptor and balance mucosal reactivity via interleukin-22. Immunity (2013) 39:372–85. 10.1016/j.immuni.2013.08.003 23973224

[106] Bessede A Gargaro M Pallotta MT Matino D Servillo G Brunacci C Aryl hydrocarbon receptor control of a disease tolerance defence pathway. Nature (2014) 511:184–90. 10.1038/nature13323 24930766 PMC4098076

[107] Goupil M Cousineau-Cote V Aumont F Senechal S Gaboury L Hanna Z Defective IL-17- and IL-22-dependent mucosal host response to *Candida albicans* determines susceptibility to oral candidiasis in mice expressing the HIV-1 transgene. BMC Immunol (2014) 15:49. 10.1186/s12865-014-0049-9 25344377 PMC4213580

[108] Guo X Qiu J Tu T Yang X Deng L Anders RA Induction of innate lymphoid cell-derived interleukin-22 by the transcription factor STAT3 mediates protection against intestinal infection. Immunity (2014) 40:25–39. 10.1016/j.immuni.2013.10.021 24412612 PMC3919552

[109] Xu X Weiss ID Zhang HH Singh SP Wynn TA Wilson MS Conventional NK cells can produce IL-22 and promote host defense in *Klebsiella pneumoniae* pneumonia. J Immunol (2014) 192:1778–86. 10.4049/jimmunol.1300039 24442439 PMC3995347

